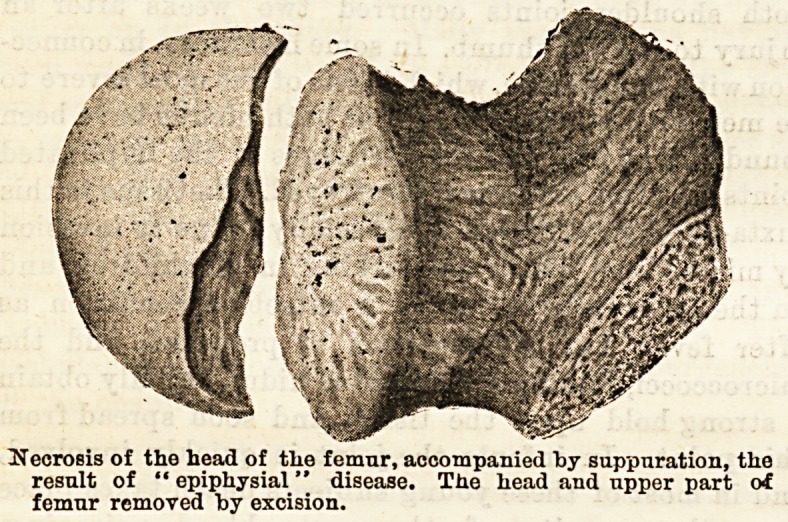# Suppurative Inflammation of Joints in Children

**Published:** 1895-04-06

**Authors:** John Poland

**Affiliations:** Surgeon to the Miller Hospital, Greenwhich


### Medical Progress and Hospital Clinics.

[*The Editor will be glad to receive offers of co-operation and contributions from members of the profession. All letters should be addressed to* The Editor, The Lodge, Porchester Square, London, W.]

### SUPPURATIVE INFLAMMATION OF JOINTS IN CHILDREN.

By John Poland, F.R.C.S., Surgeon to the Miller Hospital, Greenwich.

The condition of suppurating joints in children is one of considerable interest, not only from a diagnostic point of view, but also from that of treatment. Pus in a joint may be due to many interesting pathological states of the various structures which enter into its formation in early life. Clinically, and practically, we might classify them as originating in (1) inflammation of the synovial membrane, and (2) in inflammation of the epiphysis or periosteum (whether tuberculous, pyæmic, or from some other cause). As to the first (1) we may get pus formed in acute synovitis, and due to a variety of causes; thus it is not uncommon to find this brought about by a sprain of the soft articular structures in an unhealthy child, or even in congenital syphilis, or rickets, but the latter are rare. Punctured and other wounds penetrating the joint speedily terminate in septic inflammation and suppuration. Of all cases these demand early incision and thorough disinfection of the whole joint. An abscess in the vicinity or suppurating wound bursting into its cavity may cause the same. Another very interesting group is that due to a pyæmic general infective process in which a general arthritic condition is present. So do we frequently find suppurative synovitis following typhoid (especially at the hip joint) and scarlet fever, occasionally measles, dysentery, mumps, and, but rarely, small-pox. Gonorrheal infection, although seldom met with in children, we must include under this heading. Again, it is possible for pyæmia to arise during intra-uterine life, absorption having taken place through the umbilical cord. More than one case has been recorded in which it has arisen after birth from a septic infection during separation of the cord. Strumous synovitis, or pulpy degeneration of the synovial membrane without any bone disease, rarely suppurates nowadays unless there has been neglect or careless treatment. These affections are those over which bone-setters come to grief. In one ward I have seen three instances, treated for dislocations by the late Mr. Hutton, in which the pulpy disease by his manipulations had started acute inflammation, with suppuration of the joint. In two of these the limb had subsequently to be amputated.

Epiphysitis (2) with its inflammatory progressive results in the immediate neighbourhood of the joint, leading later to abscess of the latter, congenital syphilis, acute rickets, scurvy, struma, septicæmia, and, among local causes, such injuries as sudden jerks or sprains, blows, falls, &c., injuries during parturition from instruments, exposure to cold and wet—any of these may be sufficient to produce suppurative inflammation, whether it be acute, subacute, or chronic. The delicate new bone of the diaphysial surface of the epiphysis, the juxta-epiphysial region of Ollier, is the usual seat of the disease. Chronic epiphysial disease need not necessarily involve the joint, and is not infrequently unaccompanied by any remarkable signs, but it should be our aim to diagnose it before the joint becomes affected. Chronic epiphysitis, due to strumous disease, associated with some slight local injury to the epiphysis itself, commonly leads to suppuration; but this and other similar forms of chronic osteitic disease cannot be discussed here. Acute epiphysitis (so-called), due to pyæmic infection, is an extremely interesting form of disease, and usually attacks two or three or more epiphyses. The multiple epiphysitis leading to acute suppurative arthritis in infants has been well described by Mr. Thomas Smith, in "St. Bartholomew's Hospital Reports"—that is to say, suppuration in two or more joints in infants as young sometimes as two months; it is a question whether this might not in some have commenced *in utero*, like syphilis. As in some forms of chronic epiphysial disease, just alluded to, it usually commences in the juxta-epiphysial region, and is really a diaphysitis. The name para-epiphysitis has, therefore, been suggested for it. In illustration of this type, I would refer to the specimen described and figured in the "Pathological Transactions" for 1884, which was taken from an infant eight months old, in whom acute suppurative arthritis of both shoulder joints occurred two weeks after an injury to the left thumb. In some instances, in connection with this disease, which is one of the most severe to be met with in infancy, one or both pleuræ have been found full of pus, and the cartilages of the implicated joints are often extensively destroyed. The tissue in this juxta-epiphysial region is especially prone to invasion by microccocci, which are lurking in all children, and on the occurrence of injury or enfeebled condition, as after fever, &c., inflammation is produced, and the microccocci, finding a favourable nidus, rapidly obtain a strong hold upon the tissue, and soon spread from this point. In infants the joint is quickly involved, and in most of these young subjects death takes place from the severity of the acute blood poisoning. Analagous to this is the pyæmic arthritis often occurring in older children, and like it commencing close to the epiphysial cartilaginous disc. A good instance of this I have recorded in volume xxxviii. of the "Pathological Transactions." The affection was the result of a blow on the coracoid process of the scapula in a boy aged twelve, and was followed by acute osteo-myelitis of the scapula, suppuration of the shoulder-joint and suppuration in the epiphysis of the fourth meta-carpal bone. At the autopsy pyæmic abscesses were present in many of the viscera. In these older children acute septic osteo-myelitis of the diaphysis also frequently implicates the neighbouring joint, but fortunately in not a few (and especially in chronic osteo-myelitis) the epiphysial cartilaginous disc acts as a barrier and the joint is saved. In older children the epiphysial affection often takes the subacute type.

As an instance of the sub-acute form of epiphysial disease I might allude to the case of a boy aged ten years who had inflammation of several joints. A kick in the right groin was followed by the formation of an abscess in this situation, with high temperature and acute inflammation of the left knee and elbow joints. He appeared to get quite well, but in eight months' time acute inflammation again appeared in the left elbow with suppuration of the joint. The joint was freely opened and washed out under Listerian treatment and the child made a most perfect recovery. The movements of the elbow joint were absolutely perfect a year or two afterwards, and except for the presence of the cicatrix on each side of the olecranon it would have been impossible to say that there had been suppuration of the joint. The femoral head had, however, been destroyed and the bone gradually displaced upwards almost to the level of the iliac crest above. Another case of a girl aged ten years came under my care at the Miller Hospital. Ten weeks previously she had an alveolar abscess on the left side of the jaw followed by inflammation of the left ankle and knee joints and afterwards of the left hip. The inflammation of the ankle and knee subsided, but that of the hip-joint went on to suppuration, and the formation of an abscess in the vicinity of the great trochanter. It was suggested at the time that this was probably a case of subacute pyæmic epiphysial disease, and that probably we should find disease commencing about the neck of the bone, and possibly necrosis of the head. This was precisely the condition met with at the time of the operation—excision of the hip (see figure). The epiphysial head

Necrosis of the head of the femur, accompanied by suppuration, the result of "epiphysial" disease. The head and upper part of femur removed by excision.

of the femur was quite loose and necrosed. Probably the removal of this portion of bone would have sufficed without the graver operation of excision of the upper extremity of the bone. The child made a perfect recovery.

The signs of suppurative inflammation in a joint are fortunately well marked, at any rate, in the case of the larger articulations. The pain is severe and agonising; the temperature rises to 102 deg. or 104 deg. Tenderness, œdema, and at times redness of the skin around the swollen joint. These are often of a phlegmonous type. "Jumpings" of the limb in the case of the larger joints are often prominent signs; indeed, in many of these the least movement of the limb will cause the most intense suffering.

Fluctuation may be marked in the more acute cases, while in the chronic forms there may be but little purulent fluid in the joint, and an exploratory incision in the cases of the smaller joints is often necessary before an exact diagnosis can be arrived at. Diagnosis is made earlier at the present day, and consequently these affections of the joints are less disastrous than they were formerly. The number of excisions are fewer, amputations still less frequent. Treatment is eminently successful in a large proportion of cases. Pus in children's joints is rapid in its appearance, and as rapid in its disappearance under proper treatment. Whether it arise in the course of acute or chronic disease, it must be evacuated at once; if not, the abscess may burst and pass onwards into the limb. As soon as an abscess forms in a joint it should be treated as any other extra articular abscess and incised. A puncture with the aspirator or hypodermic needle may be first used for the purpose of confirming the diagnosis, but simple aspiration as a means of cure is not sufficient; indeed, it is dangerous. Early and free incisions are urgently necessary, and ought to be made into the joint at the most dependent parts, and the joint washed out carefully under antiseptic and aseptic precautions with 1-40 carbolic lotion, or 1-3,000 sublimate solution. This washing is very essential, although I have seen the larger joints in children do well, even in the out-patient room, without such precautions after incision. The temperature will subside, the intense pain and inflammation disappear, and often the joint may be saved without any destructive ulceration of the cartilages having taken place. Joints often become perfect in their movements, as in the elbow case just recorded. We do not and ought not to expect ankylosis as in the adult, which is the usual termination. No attempt should be made to move the joint until the wounds have entirely healed, and even then it is best to allow the patients to commence movement themselves after the removal of the splint. Appropriate treatment for supporting the strength of the patient should, of course, be adopted. Suppuration from wounds of the joint require to be freely laid open, and the articulation thoroughly cleansed in every part, each pocket of the joint being irrigated with 1-40 carbolic lotion, and drainage tubes introduced. Provided the drainage is efficient, the irrigation of the joint may be carried out with a still weaker solution of the carbolic, or 1-4,000 corrosive sublimate solution. The joint may then be closed, with the exception of the points where the drainage tubes are inserted, and antiseptic dressings applied, the limb being placed on a fixed apparatus, in the case of the knee in the straight position. At the elbow the application of the layers of antiseptic dressings are often sufficient to fix the joint in the semiflexed position. Purulent synovitis, secondary to epiphysitis, acute or chronic, requires removal of the primary cause as well as attention to the joint affection. In the acute form the epiphysis requires resection and the end of the diaphysis must be freely scraped out. The joints should be washed out with a solution of chloride of zinc and drainage tubes inserted. Even in these unpromising cases recovery may take place under prompt and energetic treatment, and the joints entirely recover their function. In acute infective osteomyelitis the joint affection is a small complication compared with the severity of the disease. In suppurating joints, in the more chronic cases of strumous children, free incision of the joint, the removal of all diseased tissue in the epiphysis or in the structures round the joint, and carefully applied antiseptic dressings with an immovable apparatus, will often avert the more serious operation of resection of the joint. Amputation is rarely necessary; it can only be called for to save the patient's life when the above measures have proved unsuccessful, and the patient's powers are evidently failing from fever, &c., in spite of the drainage.

## Figures and Tables

**Figure f1:**